# Assessment of tuberous sclerosis-associated neuropsychiatric disorders using the MINI-KID tool: a pediatric case–control study

**DOI:** 10.1186/s13023-021-01814-4

**Published:** 2021-04-17

**Authors:** Yifeng Ding, Ji Wang, Hao Zhou, Taoli Li, Shuizhen Zhou, Yi Wang

**Affiliations:** 1 Department of Neurology, Children’s Hospital of Fudan University, National Children’s Medical Center, No. 399 Wanyuan Road, Minhang District, Shanghai, 201102 China; 2 Department of Pediatrics, Guizhou Provincial People’s Hospital, Medical College of Guizhou University, Guiyang, China; 3 Department of Neurology, Xi’an Children’s Hospital, Xi’an, 710003 China

**Keywords:** TSC, TAND, Child, MINI-KID, ADHD, Psychiatric disorders

## Abstract

**Background:**

The tuberous sclerosis-associated neuropsychiatric disorders (TAND) have not previously been studied in China. We aimed to assess the psychiatric level of individuals with TAND using the Mini International Neuropsychiatric Interview for Children (MINI-KID) in China.

**Results:**

A total of 83.16% of individuals (79/95) had at least one TAND, and 70.53% (67/95) had an intellectual disability. The MINI-KID tool diagnosed 16 neuropsychiatric diseases, the most common of which were attention-deficit/hyperactivity disorder (ADHD) (51.58%, 49/95) and social anxiety disorder (30.53%, 29/95). The number of children with psychiatric diseases in the tuberous sclerosis complex (TSC) group was significantly greater than the number in the typically developing group (*P* < 0.0001). Notably, 69.47% (66/95) had two or more psychiatric disorders. Pervasive developmental disorder (PDD) was often co-morbid with other psychiatric disorders.

**Conclusions:**

This study used the structured and systematic MINI-KID scale to determine the diagnosis of psychiatric co-morbidities in a relatively large sample, suggesting a higher rate. By comparing the status of individuals with TSC with typically developing children, the results suggests that neuropsychiatric co-morbidities are significantly higher in individuals with TSC. Research has revealed the frequent presence of two, three or more neuropsychiatric diseases in individuals with TSC.

**Supplementary Information:**

The online version contains supplementary material available at 10.1186/s13023-021-01814-4.

## Background

Tuberous sclerosis complex (TSC) is an autosomal dominant genetic disease characterized by the formation of hamartomas in multiple organ systems with various symptoms and degrees of severity. The currently known pathogenic genes are the *TSC1* (9q34) and *TSC2* (16p13.3) genes [1]. In addition to the physical manifestations in multiple organs, children with TSC may also suffer from a wide array of neurodevelopmental, behavioral, psychiatric, and psychosocial difficulties. The Neuropsychiatry Panel proposed the term TSC-associated neuropsychiatric disorders (TAND) as an umbrella term to include the full range of mental health issues. The panel defined six levels of investigation: behavioral, psychiatric, intellectual, academic, neuropsychological, and psychosocial levels [2, 3] Neurological and psychiatric complications and kidney disease are often the greatest burdens imposed by TSC, but TAND have not received enough attention [4–6]. A 2010 survey of members of the British Tuberous Sclerosis Association revealed that only 18% of individuals with TSC had been assessed or treated for neuropsychiatric disorders [7]. With the exception of the TOSCA study investigating a larger sample size of 1400 children (possibly including some Chinese pediatric participants) [8], very few studies have assessed the rate and pattern of TAND in children with TSC and none have been reported in China [9].

As a simple, reliable and effective interview, the MINI-International Neuropsychiatric Interview for Children (MINI-KID) was developed based on Diagnostic and Statistical Manual of Mental Disorders, 4th edition (DSM-IV) and International Classification of Disease, 10th version (ICD-10) criteria for the assessment and diagnosis of psychiatric disorders [10]. The study uses a structured tool (MINI-KID), intelligence quotient (IQ) measures and typically developing control sample to outline the profile of psychiatric disorders in children and adolescents with TSC [10–13]. This study will provide a systematic assessment of the rate of psychiatric disorders in children with TSC.

## Methods

### Study population

This study was carried out from September 2019 to November 2019. Children with TSC were recruited from among all individuals with TSC aged 6–16 years who were treated in the Department of Neurology at the Children’s Hospital of Fudan University, National Children’s Medical Center. All individuals met the latest diagnostic criteria for tuberous sclerosis [2] and were examined for mutations in the *TSC1/TSC2* genes. The exclusion criterion was refusal to participate.

We also recruited children with typical development as the control group in the health examination center of Children’s Hospital of Fudan University and matched them by age and sex. The criteria for children with typical development were as follows: (1) typical motor and language development; (2) no history of seizures or other neurological diseases; (3) no other chronic diseases (such as diabetes, asthma or cancer); and (4) no first-degree relatives with a family history of mental illness. The exclusion criterion was parental refusal to provide informed consent. Children with typical development were screened by the deputy chief physician and attending physician of the neurology department, who each had more than 5 years of experience.

Written informed consent was received from all participating families. This study was approved by the ethics committee of Children’s Hospital of Fudan University.

### Data collection

#### Standardized questionnaire

After providing informed consent, parents or guardians were invited to participate in interviews, and standardized questionnaires were used to obtain demographic and clinical data. The demographic data collected were the date of birth, sex, ethnicity, parental education level, family income, and residence. The cognitive assessment was conducted using the Wechsler Intelligence Scale for Children in Chinese (WISC-C). The clinical data collected were growth and development, family history, age at onset of epilepsy, duration of epilepsy, current number of antiepileptic drugs, frequency of seizures, spasm, and TSC clinical manifestations.

#### MINI-KID (parent version)

Parents or guardians of the children with TSC were interviewed using the MINI-KID (parent version) for children and adolescents. The Chinese version of the MINI-KID (parent version) 5.0 was translated by Liu Y.X. and other researchers from Peking University Institute of Mental Health in 2010. The study by Liu Y.X. and other studies have confirmed that the Chinese version of the MINI-KID (parent version) has good reliability and validity [14] and that the parent version is more sensitive than the child version [15, 16].

The MINI-KID is a structured diagnostic scale designed according to the DSM-IV and the ICD-10. The scale has 25 modules to diagnose 24 mental illnesses and suicidal tendencies in children and adolescents between the ages of 6 and 16 years [10, 13]. Each module includes a screening questionnaire and a diagnostic questionnaire. The MINI-KID was administered by highly trained neurologists (minimum 5 years of experience in the diagnosis of neuropsychiatric diseases in children) in this study, and the diagnosis was based solely on whether the responses to the screening questions were positive. All questions had a “yes/no” response format [10, 13]. The assessment took approximately 30–45 min to complete. As a preliminary exploration of MINI-KID in the context of TAND, our diagnosis was based on whether the diagnostic criteria of MINI-KID were met, and therefore some of the results may not reflect final diagnoses provided by expert psychiatric evaluations.

All parents of children participating in this study were administered the questionnaire face-to-face after signing the informed consent form.

### Statistical analysis

Continuous variables are presented as the means and standard deviations (SD). Continuous variables were analyzed using the ANOVA F-test, and categorical variables were analyzed using chi-square or Fisher's exact tests. Multivariate logistic regression models were performed with each TAND risk factors as an independent variable. Those known risk or protective factors for TAND were included in our regression analysis. We adjusted for sex (male/female), maternal education (years) (≤ 9/9–12/ > 12), paternal education (years) (≤ 9/9–12/ > 12), family income (RMB) (< 5000/5000–10,000/ > 10,000) and residence (suburban or rural/urban) in the adjusted model. A two-sided *P* value < 0.05 was statistically significant. All analyses were performed using JMP Pro 15.0.0 software (version 15.0.0, SAS Institute Inc., USA).

## Results

### Characteristics of the study population

Our TSC cohort included 291 individuals, of which 117 (40.21%) were 6–16 years old. Sixteen people declined to participate and 86.32% (101/117) parents signed the informed consent form, but 6 individuals did not complete all the questionnaires (withdrew halfway). Ninety-five individuals (81.20%, 95/117) completed the questionnaire, including 46 females (48.42%) and 49 males (51.58%), whose average age was 10.02 ± 3.10 years. All participants were of the Han ethnicity (Table 1). In addition, 95 children with typical development (males:females = 51:44, age 10.3 ± 2.4 years) were recruited (Table 1).

**Table 1:** Sociodemographic comparison of patients with TSC and normal controls

Sociodemographic feature	TSC	NC	χ^2^/F	*P* value
N	95	95		
Male	49 (51.58)	51 (53.68)	0.08	0.17
Age at interview (years)	10.12 ± 3.10	10.32 ± 2.41	1.66	0.36
Paternal education (years)			0.33	0.72
≤ 9	35 (36.84)	35 (36.84)		
9–12	20 (21.05)	23 (24.21)		
> 12	40 (42.11)	37 (38.95)		
Maternal education (years)			2.75	0.57
≤ 9	38 (40.00)	30 (31.58)		
9–12	18 (18.95)	27 (28.42)		
> 12	39 (41.05)	38 (40.00)		
Family income (RMB)			3.42	0.09
< 5000	16 (16.84)	5 (5.26)		
5000–10,000	51 (53.68)	63 (66.32)		
> 10,000	28 (29.47)	27 (28.42)		
Residence			1.37	0.11
Suburban or rural	50 (52.63)	58 (61.05)		
Urban	45 (47.37)	37 (38.95)		

Data are presented as means ± SD and n (%)

ANOVA F-test was performed for continuous variables and the chi-square test was performed for categorical values

*TSC* tuberous sclerosis complex, *NC* normal controls (children with normal nervous system development)

All 95 children were genetically tested, and the *TSC1*:*TSC2*:NMI (no mutation identified) ratio was 27:58:10. More sporadic cases were identified than familial cases (n = 55 VS n = 40). Sixty-seven children had an intellectual disability (ID) (IQ ≤ 70) (70.53%), 34 (35.79%) had mild to moderate ID (IQ 35–70), and 33 (34.74%) had severe ID (IQ < 35). Of the 95 children, 76 had a history of epilepsy (80.00%), and 49 of the individuals with TSC and epilepsy developed drug-resistant epilepsy (64.47%). The average age at seizure onset was 2.50 ± 3.59 years, and the duration was 7.65 ± 4.37 years. Eighty-seven individuals had cortical tubers, 86 children had subependymal nodules (SENs), and 3 individuals had subependymal giant cell astrocytoma (SEGA).

### Rate of neuropsychiatric disorders in individuals with TSC and typically developing controls

Seventy-nine (83.16%) children with TSC developed TAND. Among children with TAND, the male: female ratio was 41:38. Sixteen neuropsychiatric diseases were diagnosed, the most common of which was attention-deficit/hyperactivity disorder (ADHD) (51.58%, 49/95), followed by social anxiety disorder (30.53%, 29/95), panic disorder (26.32%, 25/95), specific phobia (26.32%, 25/95), pervasive developmental disorder (PDD) (22.11%, 21/95), (mild) manic episodes (22.11%, 21/95), agoraphobia (16.84%, 16/95), tic disorder (15.79%, 15/95) and separation anxiety disorder (10.53%, 10/95) (Additional file 1: Table S1). The rates of ADHD, social anxiety disorder, panic disorder, specific phobia, PDD, (mild) manic episodes, agoraphobia, tic disorder, separation anxiety disorder, major depressive episode, suicide and obsessive–compulsive disorder were significantly different between the TSC and control groups (*P* < 0.05) (Additional file 1: Table S1).

**Fig. 1:**
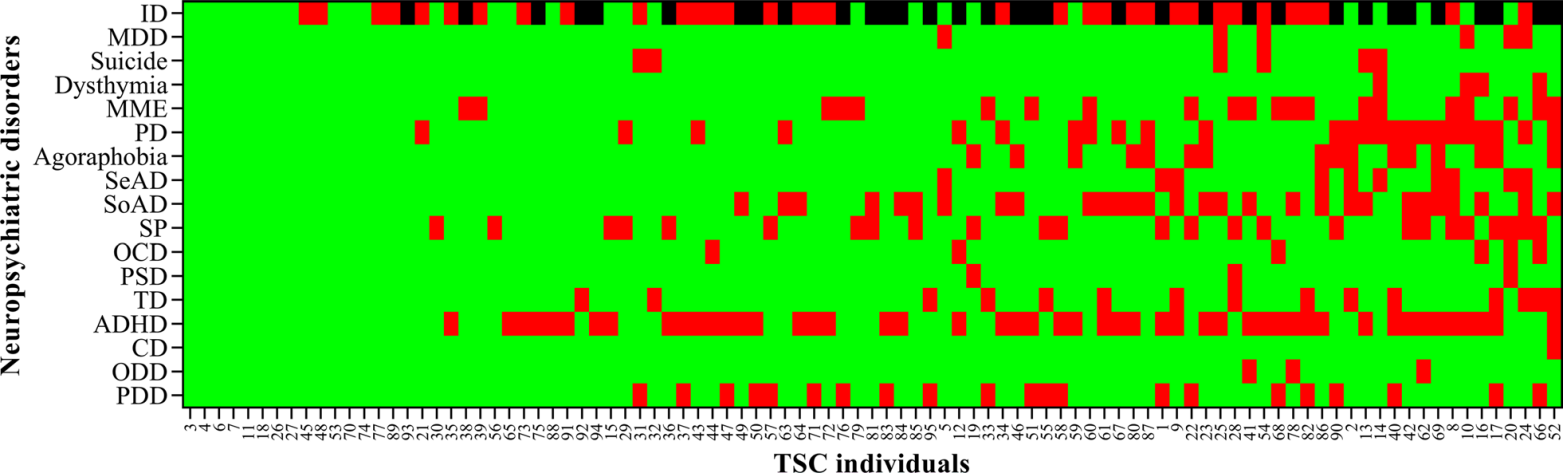
Heat map analysis of 16 psychiatric disorders in 95 individuals. Among the 16 mental disorders, red = “yes”, green = “no”; among the intellectual developmental disorders, green = “no ID”, red = “mild to moderate ID”, and black = “severe ID”. *ID* intellectual disability, *MDD* major depressive episode, *MME* (mild) manic episodes, *PD* panic disorder, *SeAD* separation anxiety disorder, *SoAD* social anxiety disorder, *SP* specific phobia, *OCD* obsessive–compulsive disorder, *PSD* posttraumatic stress disorder, *TD* tic disorder, *ADHD* attention-deficit/hyperactivity disorder, *CD* conduct disorder, *ODD* oppositional defiant disorder, *PDD* attention-deficit/hyperactivity disorder

Among individuals with TSC, 69.47% had two or more TAND. The rates of psychiatric disorders in participants with different levels of ID are shown in Table 2. Except for social anxiety disorder, ADHD and PDD, no significant difference in the prevalence of neuropsychiatric disorders was observed after stratification by intellectual development level. The co-morbidity of neuropsychiatric disorders for each individual is presented as a heat map (Fig. 1). The heat map presents these disorders in individuals with different levels of intellectual development. PDD was often co-morbid with other diseases (one, two and three co-morbidities in nine, four and five participants, respectively). The most frequent co-morbidities included ADHD, tic disorder, specific phobia and (mild) manic episodes.

**Table 2:** Distribution of neuropsychiatric disorders in individuals with different levels of ID

Neuropsychiatric disorders	Different levels of ID			*P* value
	Normal (n = 28)	Mild-moderate (n = 34)	Severe-profound (n = 33)	
Major depressive episode	3 (10.71)	3 (8.82)	0 (0.00)	0.17
Suicide	2 (7.14)	3 (8.82)	1 (3.03)	0.61
Dysthymia	2 (7.14)	0 (0.00)	2 (6.06)	0.31
(Mild) manic episodes	4 (14.29)	8 (23.53)	9 (27.27)	0.46
Panic disorder	6 (21.43)	7 (20.59)	12 (36.36)	0.27
Agoraphobia	3 (10.71)	4 (11.76)	9 (27.27)	0.14
Separation anxiety disorder	4 (14.29)	4 (11.76)	2 (6.06)	0.56
Social anxiety disorder	4 (14.29)	15 (44.12)	20 (60.61)	0.01
Specific phobia	10 (35.71)	7 (20.59)	8 (24.24)	0.38
Obsessive–compulsive disorder	1 (3.57)	1 (2.94)	4 (12.12)	0.24
Posttraumatic stress disorder	2 (7.14)	1 (2.94)	0 (0.00)	0.28
Tic disorder	2 (7.14)	5 (14.71)	8 (24.24)	0.18
ADHD	6 (21.43)	21 (61.76)	22 (66.67)	0.01
Conduct disorder	0 (0.00)	0 (0.00)	1 (3.03)	0.39
Oppositional defiant disorder	1 (3.57)	3 (8.82)	3 (9.09)	0.98
Pervasive developmental disorder	0 (0.00)	8 (23.53)	13 (39.39)	0.01

Data are presented as n (%)

*ID* intellectual development, *ADHD* attention-deficit/hyperactivity disorder

A total of 85.19% (23/27) of individuals in the *TSC1* gene mutation group, 84.48% (49/58) in the *TSC2* gene mutation group and 70.00% (7/10) in the NMI group had neuropsychiatric disorders, but the difference was not significant (*P* = 0.546). The rates of psychiatric disorders in individuals with different genotypes are shown in Additional file 2: Table S2.

### Analysis of TAND-related risk factors

Ninety-five children with TSC were divided into the TAND group (n = 79) and without TAND group (n = 16). After adjustment for sex, parents’ education, family income (RMB) (< 5000/5000–10,000/ > 10,000) and residence, the multivariate logistic regression models found that an earlier age of onset (< 2 years) (*P* = 0.03), more frequent seizure frequency (more than once a month) (*P* = 0.04) and use of a greater number of antiepileptic drugs (≥ 2) (*P* = 0.04) were closely related to the occurrence of TAND (Additional file 3: Table S3, Additional file 4: Table S4). No significant correlation was observed with neoplastic diseases (RAML, LAM and cardiac rhabdomyomas), cortical tubers, SENs, or SEGA (*P* > 0.05) (Additional file 3: Table S3).

## Discussion

To our knowledge, this cross-sectional study of TAND using the MINI-KID is the first to be conducted, and it is also the TAND cohort study with the largest sample performed in China. The rate of neuropsychiatric disorders in children with TSC is significantly higher than in individuals with typical neurodevelopment. Consistent with previous studies, TAND is present in childhood and adolescence [3, 5, 17, 18]. The lifetime prevalence of TAND is approximately 90% [3]. Anxiety, depression, and ADHD are more common in individuals with TSC [18]. In our cohort, 77 children with TSC had neuropsychiatric disorders, for a rate of 81.05%, which far exceeded our expectations. However, clinicians pay far less attention to mental illnesses than neurological disorders (such as epilepsy and SEGA). As reported in previous studies, less than 40% of the more than 2000 individuals from 31 countries underwent an intelligence assessment, and the proportion of missing TAND data is high. Thus, TAND are not adequately identified and processed, even at TSC specialty centers [18]. Our study suggests that the disease burden of TSC is far greater than previously realized, which will serve as a basis for a better distribution of medical resources.

According to Prather and de Vries et al. [19, 20], autism spectrum disorder (ASD) and ADHD are the most common TAND in children, and anxiety/mood disorders are the most common in adults. The incidence of ADHD has varied from 30 to 60%, based on the study method and diagnostic criteria [21–24]. The MINI-KID serves as a short, standardized, universal assessment tool to systematically assess parent reports of health-related neuropsychiatric disorders in children with chronic diseases [25–27]. To our knowledge, this report is the first application of the MINI-KID tool in a TSC cohort to accurately screen and diagnose TAND. We found that ADHD, social anxiety disorder, panic disorder and specific phobia were the most common disorders in this group. The rate of ADHD in our cohort was significantly higher than that in the TOSCA cohort (51.58% vs. 22.4%) [8], and we suggest several possible explanations below. (1) The age distribution of children in the TOSCA cohort was 0–18 years old, while the age distribution of children in our cohort was 6–16 years old, consistent with the high rate age of ADHD diagnosis. (2) Our cohort included individuals with TSC from all over the country. In some areas, a comprehensive and systematic treatment standard has not been established, and clinicians do not even know enough about TAND to intervene in a timely manner. (3) Unlike the TAND Checklist method adopted by the TOSCA study, the MINI-KID tool was used for both screening and diagnosis in our cohort.

Although the rate of anxiety was very high in previous cohorts [8, 17, 18], the diagnosis rate of anxiety was lower in our study (25.80–56.00% vs. 10.53%), while the rate of depression was approximately the same (8.20% vs. 6.32%). In the future, clinicians should focus on screening children for emotional disorders and anxiety responses. Our results are comparable to those of a cohort study of 32 samples in Italy [9].

Interestingly, in our study, we identified a number of psychiatric disorders that were uncommon or unreported in previous TAND studies, such as (mild) manic episodes, oppositional defiant disorder, and obsessive–compulsive disorder. We found and reported this phenomenon. We did not consider PDD as a confounder because these individuals had relevant clinical manifestations and did not meet the diagnostic criteria for PDD in the MINI-KID. These disorders are rare but had a sufficiently high rate to draw our attention to psychiatric disorders other than the common TAND.

We sought to identify the risk factors associated with the occurrence of TAND and found that an earlier onset age (< 2 years), a longer seizure duration (≥ 2 years), more frequent seizures (more than 1 seizure per month), and the use of more antiepileptic drugs (≥ 2) are closely related to the occurrence of TAND. These findings are similar to previous studies [8, 17, 19].

In view of the high prevalence of TAND in children with TSC, this study focused on drawing the attention of pediatric neurologists to TAND for the early screening and diagnosis of neuropsychiatric disorders. Individuals with a confirmed diagnosis will be referred to the relevant psychiatrist.

The Neuropsychiatry Panel offers the TAND Checklist, a simple framework for a conversation about TAND [3], to make TAND screening easy and convenient. It is a screening tool (not a diagnostic tool like the MINI-KID) that assesses all levels of TAND (not only psychiatric). It would therefore be of potential value to work with the developers of the TAND Checklist to translate and authorize the TAND Checklist for use in Chinese populations, and a potentially very informative approach would be to use the MINI-KID to validate the psychiatric level of the TAND Checklist using the Chinese sample presented here.

This study has several limitations. MINI-KID is a classification, not a comprehensive diagnosis; it is not based on ICD-11/DSM-5. It does not group co-occurring conditions and does not have a hierarchy for diagnostic classification. It only examines the psychiatric level of TAND and not the many other levels as outlined in the TAND construct and TAND Checklist. The present study was a cross-sectional study of a single center, and future multicenter prospective studies are needed to further verify the results. In addition, this study used only one method to explore the mental health of children with TSC and did not use the TAND Checklist for comparison. The study population included children aged 6–16 years and excluded younger children. At the National Children’s Medical Center, we may have a biased sample of children with TSC (higher prevalence of epilepsy and drug-refractory epilepsy, and altered intellectual development).

## Conclusions

This study used the structured and systematic MINI-KID scale to determine the diagnosis of psychiatric co-morbidities in a relatively large sample, suggesting their higher rate. By comparing the status of individuals with TSC with typically developing children, the results suggest a significantly higher rate of neuropsychiatric co-morbidities in individuals with TSC. Moreover, individuals with TSC often present with two, three or more neuropsychiatric diseases.

## Supplementary Information

**Additional file 1.** Distribution of neuropsychiatric disorders in individuals with TSC and typically developing controls.

**Additional file 2.** Distribution of neuropsychiatric disorders in individuals with different genotypes.

**Additional file 3.** Analysis of factors associated with the TAND incidence.

**Additional file 4.** Risk factors associated with TAND.

## Data Availability

The datasets used during and/or analyzed during the current study are available from the corresponding author upon request.

## Abbreviations

TSC: Tuberous sclerosis complex
TAND: Tuberous sclerosis-associated neuropsychiatric disorders
MINI-KID: Mini International Neuropsychiatric Interview for Children
ID: Intellectual disability
NMI: No mutation identified
SENs: Subependymal nodules
SEGA: Subependymal giant cell astrocytoma
RAML: Renal angiomyolipoma
LAM: Lymphangioleiomyomatosis
PDD: Pervasive developmental disorder
ADHD: Attention-deficit/hyperactivity disorder
AED: Antiepileptic drugs
ASD: Autistic spectrum disorder
