# Supplementary material for: Assessment of tuberous sclerosis-associated neuropsychiatric disorders using the MINI-KID tool: a pediatric case–control study
Source: Orphanet J Rare Dis. 2021 Apr 17;16:181. doi: 10.1186/s13023-021-01814-4 (PMC8052770; doi:10.1186/s13023-021-01814-4)
Supplement: Supplementary file 1 — Additional file 1. Distribution of neuropsychiatric disorders in individuals with TSC and typically developing controls. [file 13023_2021_1814_MOESM1_ESM.docx]

Additional file 1: Table S1. Distribution of neuropsychiatric disorders in individuals with TSC and typically developing controls

| Neuropsychiatric disorders | TSC | Controls | *P* value |
| --- | --- | --- | --- |
| N | 95 | 95 |  |
| ADHD | 49 (51.58) | 6 (6.32) | <0.01 |
| Inattentive type | 27 (28.43) | 3 (3.16) |  |
| Combined type | 13 (13.68) | 2 (2.11) |  |
| Hyperactive impulsive | 9 (9.47) | 1 (1.05) |  |
| Social anxiety disorder | 29 (30.53) | 2 (2.11) | <0.01 |
| Panic disorder | 25 (26.32) | 0 (0.00) | <0.01 |
| Specific phobia | 25 (26.32) | 1 (1.05) | <0.01 |
| Pervasive developmental disorder | 21 (22.11) | 0 (0.00) | <0.01 |
| (Mild) manic episodes | 21 (22.11) | 0 (0.00) | <0.01 |
| Agoraphobia | 16 (16.84) | 0 (0.00) | <0.01 |
| Tic disorder | 15 (15.79) | 0 (0.00) | <0.01 |
| Separation anxiety disorder | 10 (10.53) | 1 (1.05) | 0.01 |
| Oppositional defiant disorder | 7 (7.37) | 1 (1.05) | 0.07 |
| Major depressive episode | 6 (6.32) | 0 (0.00) | 0.03 |
| Suicide | 6 (6.32) | 0 (0.00) | 0.03 |
| Obsessive-compulsive disorder | 6 (6.32) | 0 (0.00) | 0.03 |
| Dysthymia | 4 (4.21) | 0 (0.00) | 0.12 |
| Posttraumatic stress disorder | 3 (3.16) | 0 (0.00) | 0.25 |
| Conduct disorder | 1 (1.05) | 0 (0.00) | 0.32 |
| No neuropsychiatric disorders | 16 (16.84) | 78 (82.11) | <0.0001 |

ADHD: attention-deficit/hyperactivity disorder;

Data are presented as n (%). Chi-square or Fisher’s exact tests were used to analyze categorical values.
