# Supplementary material for: Assessment of tuberous sclerosis-associated neuropsychiatric disorders using the MINI-KID tool: a pediatric case–control study
Source: Orphanet J Rare Dis. 2021 Apr 17;16:181. doi: 10.1186/s13023-021-01814-4 (PMC8052770; doi:10.1186/s13023-021-01814-4)
Supplement: Supplementary file 2 — Additional file 2. Distribution of neuropsychiatric disorders in individuals with different genotypes. [file 13023_2021_1814_MOESM2_ESM.docx]

Additional file 2: Table S2. Distribution of neuropsychiatric disorders in individuals with different genotypes

| Neuropsychiatric disorders | NMI (n=10) | *TSC1* (n=27) | *TSC2* (n=58) |
| --- | --- | --- | --- |
| Major depressive episode | 0 (0.00) | 3 (11.11) | 3 (5.17) |
| Suicide | 1 (10.00) | 2 (7.40) | 3 (5.17) |
| Dysthymia | 1 (10.00) | 2 (7.40) | 1 (1.72) |
| (Mild) manic episodes | 2 (20.00) | 6 (22.22) | 13 (22.41) |
| Panic disorder | 0 (0.00) | 7 (25.93) | 18 (31.03) |
| Agoraphobia | 0 (0.00) | 5 (18.52) | 11 (18.97) |
| Separation anxiety disorder | 0 (0.00) | 4 (14.81) | 6 (10.34) |
| Social anxiety disorder | 2 (20.00) | 8 (29.63) | 29 (50.00) |
| Specific phobia | 3 (30.00) | 9 (33.33) | 13 (22.41) |
| Obsessive-compulsive disorder | 1 (10.00) | 2 (7.40) | 3 (5.17) |
| Posttraumatic stress disorder | 0 (0.00) | 1 (3.70) | 2 (3.45) |
| Tic disorder | 2 (20.00) | 2 (7.41) | 11 (18.97) |
| ADHD | 3 (30.00) | 13 (48.15) | 33 (56.89) |
| Conduct disorder | 0 (0.00) | 0 (0.00) | 1 (1.72) |
| Oppositional defiant disorder | 1 (10.00) | 3 (11.11) | 3 (5.17) |
| Pervasive developmental disorder | 2 (20.00) | 6 (22.22) | 13 (22.41) |

NMI: no mutation identified; ADHD: attention-deficit/hyperactivity disorder

Data are presented as n (%).
