# Supplementary material for: Assessment of tuberous sclerosis-associated neuropsychiatric disorders using the MINI-KID tool: a pediatric case–control study
Source: Orphanet J Rare Dis. 2021 Apr 17;16:181. doi: 10.1186/s13023-021-01814-4 (PMC8052770; doi:10.1186/s13023-021-01814-4)
Supplement: Supplementary file 3 — Additional file 3. Analysis of factors associated with the TAND incidence. [file 13023_2021_1814_MOESM3_ESM.docx]

Additional file 3: Table S3. Analysis of factors associated with the TAND incidence

| Predictor | TAND | Without TAND | *P* value |
| --- | --- | --- | --- |
| N | 77 | 18 |  |
| Male | 39 (50.65) | 10 (55.56) | 0.71 |
| Paternal education (years) |  |  | 0.74 |
| ≤9 | 29 (37.66) | 6 (33.33) |  |
| 9-12 | 17 (20.08) | 3 (16.67) |  |
| >12 | 31 (40.26) | 9 (50.00) |  |
| Maternal education (years) |  |  | 0.94 |
| ≤9 | 31 (40.26) | 7 (38.89) |  |
| 9-12 | 15 (19.48) | 3 (16.67) |  |
| >12 | 31 (40.26) | 8 (44.44) |  |
| Family income (RMB) |  |  | 0.36 |
| <5000 | 11 (14.29) | 5 (27.78) |  |
| 5000-10000 | 42 (54.55) | 9 (50.00) |  |
| >10000 | 24 (31.17) | 4 (22.22) |  |
| Residence |  |  | 0.07 |
| Suburban or rural | 37 (48.05) | 13 (72.22) |  |
| Urban | 40 (51.95) | 5 (27.78) |  |
| Epilepsy | 64 (83.12) | 12 (66.67) | 0.12 |
| Age at seizure onset (<2 years) | 45 (58.44) | 5 (27.78) | 0.03 |
| Seizure frequency ≥ 1/month | 30 (38.96) | 2 (11.11) | 0.04 |
| Spasm | 20 (25.97) | 1 (5.56) | 0.14 |
| Gene |  |  | 0.50 |
| NMI | 7 (9.09) | 3 (16.67) |  |
| *TSC1* | 23 (29.87) | 4 (22.22) |  |
| *TSC2* | 47 (61.04) | 11 (61.11) |  |
| Family history | 36 (46.75) | 4 (22.22) | 0.13 |
| Mutation type |  |  | 0.51 |
| Nonsense | 20 (25.97) | 6 (33.33) |  |
| Missense | 16 (20.78) | 3 (16.67) |  |
| Frame shift | 21 (27.27) | 3 (16.67) |  |
| Splicing | 7 (9.09) | 2 (11.11) |  |
| Small deletion | 2 (2.60) | 1 (5.56) |  |
| Large deletion | 4 (5.19) | 0 (0) |  |
| Polytherapy（≥2 AEDs） | 55 (71.43) | 8 (44.44) | 0.04 |
| Hypomelanotic macules | 72 (93.51) | 18 (100.00) | 0.30 |
| Angiofibromas | 32 (41.56) | 8 (44.44) | 0.48 |
| Shagreen patches | 39 (50.65) | 12 (66.67) | 0.19 |
| Ungual fibromas | 7 (9.09) | 0 (0) | 0.22 |
| RAMLs | 52 (67.53) | 13 (72.22) | 0.98 |
| Cardiac rhabdomyomas | 64 (83.12) | 14 (77.78) | 0.42 |
| LAM | 17 (22.08) | 7 (38.89) | 0.06 |
| SENs | 69 (89.61) | 17 (94.44) | 0.63 |
| Cortical tubers | 70 (90.91) | 17 (94.44) | 0.73 |
| SEGA | 3 (3.90) | 0 (0) | 0.43 |

TAND: tuberous sclerosis-associated neuropsychiatric disorders; TSC: tuberous sclerosis complex;

AED: antiepileptic drugs; NMI: no mutation identified; RAML: renal angiomyolipoma; LAM: lymphangioleiomyomatosis; SENs: subependymal nodules; SEGA: subependymal giant cell astrocytoma

Data are presented as n (%). Logistic regression models were used to explore the risk factors for TAND.
