# Supplementary material for: Assessment of tuberous sclerosis-associated neuropsychiatric disorders using the MINI-KID tool: a pediatric case–control study
Source: Orphanet J Rare Dis. 2021 Apr 17;16:181. doi: 10.1186/s13023-021-01814-4 (PMC8052770; doi:10.1186/s13023-021-01814-4)
Supplement: Supplementary file 4 — Additional file 4. Risk factors associated with TAND. [file 13023_2021_1814_MOESM4_ESM.docx]

Additional file 4: Table S4. Risk factors associated with TAND

| Predictor | TAND | Crude | Adjusted |
| --- | --- | --- | --- |
|  |  | OR (95% CI) | OR (95% CI)* |
| Age at seizure onset |  |  |  |
| >2 years | 33 (41.77) | Ref. | Ref. |
| <2 years | 46 (58.23) | 4.18 (1.24, 14.12) | 6.53 (1.16, 36.80) |
| Seizure frequency |  |  |  |
| < 1/month | 49 (62.02) | Ref. | Ref. |
| ≥ 1/month | 30 (37.98) | 9.18 (1.15, 73.11) | 21.28 (1.14, 59.24) |
| Polytherapy |  |  |  |
| <2 AED | 42 (53.17) | Ref. | Ref. |
| ≥2 AEDs | 37 (46.84) | 3.82 (1.01, 14.45) | 15.28 (1.65, 41.64) |

TAND: tuberous sclerosis-associated neuropsychiatric disorders

Data are presented as n (%).

*: adjusted for sex (male/female), maternal education (years) (≤9/9-12/>12), paternal education (years) (≤9/9-12/>12), family income (RMB) (<5000/5000-10000/>10000) and residence (suburban or rural/urban).
